# Genetic engineering of *Pseudomonas chlororaphis* Lzh-T5 to enhance production of trans-2,3-dihydro-3-hydroxyanthranilic acid

**DOI:** 10.1038/s41598-021-94674-8

**Published:** 2021-08-12

**Authors:** Kaiquan Liu, Ling Li, Wentao Yao, Wei Wang, Yujie Huang, Ruiming Wang, Piwu Li

**Affiliations:** 1 State Key Laboratory of Biobased Material and Green Papermaking (LBMP), School of Bioengineering, Qilu University of Technology (Shandong Academy of Sciences), Jinan, 250353 Shandong People’s Republic of China; 2 Shandong Provincial Key Laboratory of Applied Microbiology, Ecology Institute, Qilu University of Technology (Shandong Academy of Sciences), Jinan, 250103 People’s Republic of China; 3 State Key Laboratory of Microbial Metabolism, School of Life Sciences and Biotechnology, Shanghai Jiao Tong University, Shanghai, 200240 People’s Republic of China

**Keywords:** Metabolic engineering, Applied microbiology

## Abstract

Trans-2,3-dihydro-3-hydroxyanthranilic acid (DHHA) is a cyclic β-amino acid used for the synthesis of non-natural peptides and chiral materials. And it is an intermediate product of phenazine production in *Pseudomonas spp*. Lzh-T5 is a *P. chlororaphis* strain isolated from tomato rhizosphere found in China. It can synthesize three antifungal phenazine compounds. Disruption the *phzF* gene of *P. chlororaphis* Lzh-T5 results in DHHA accumulation. Several strategies were used to improve production of DHHA: enhancing the shikimate pathway by overexpression, knocking out negative regulatory genes, and adding metal ions to the medium. In this study, three regulatory genes (*psrA*, *pykF,* and *rpeA*) were disrupted in the genome of *P. chlororaphis* Lzh-T5, yielding 5.52 g/L of DHHA. When six key genes selected from the shikimate, pentose phosphate, and gluconeogenesis pathways were overexpressed, the yield of DHHA increased to 7.89 g/L. Lastly, a different concentration of Fe^3+^ was added to the medium for DHHA fermentation. This genetically engineered strain increased the DHHA production to 10.45 g/L. According to our result, *P. chlororaphis* Lzh-T5 could be modified as a microbial factory to produce DHHA. This study laid a good foundation for the future industrial production and application of DHHA.

## Introduction

Trans 2,3-dihydro-3-hydroxyanthranilic acid (DHHA) can be used in cycloaddition reactions as an enantiomerically pure building block, for biosynthesis of unnatural peptides, and for preparation of various useful intermediate acid derivatives of benzoic acid, such as 3-hydroxyanthranilic acid and anthranilic acid, which are important aromatic compounds^1–4^. These compounds are widely used in chemicals, food, cosmetics, and pharmaceuticals. In the current market, production of aromatic compounds relies heavily on direct extraction from plants or petroleum-derived chemical processes. The demand for establishing new sustainable sources and renewable aromatics has increased rapidly in recent years. The sustainable production of aromatics has drawn great interest^5–8^. Microbial bioproduction using abundant feedstocks is a highly promising alternative^8^.

**Figure 1:**
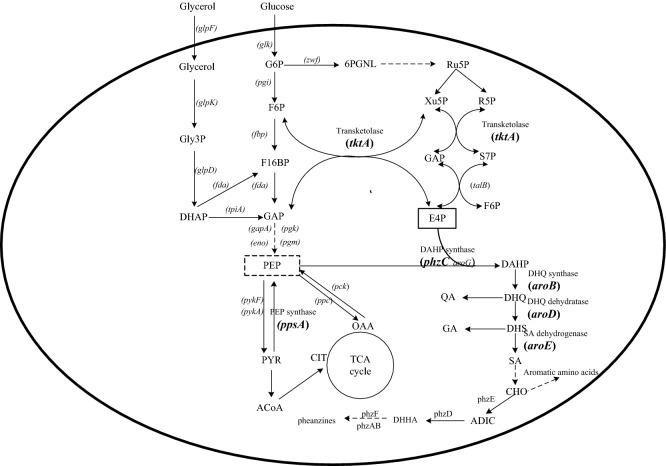
Central carbon metabolism related to the biosynthesis of phenazines in *P. chroloraphis* Lzh-T5. The enzymes: *pgi*, phosphoglucose isomerase; *glk*, glucokinase; *eno*, enolase; *gapA*, glyceraldehyde 3-phosphate dehydrogenase; *glpK*, glycerol kinase; *glpF*, glycerol facilitator; *fda*, fructose-1,6-P2 aldolase; *glpD*, glycerol-3-P dehydrogenase; *fbp*, fructose 1,6-bisphosphatase; *tpiA*, triosephosphate isomerase; *talB*, transaldolase; *zwf*, G6P dehydrogenase; *pck*, PEP carboxykinase; *ppc*, PEP carboxylase; *pgm*, phosphoglyceromutase; *pgk*, phosphoglycerate kinase; *phzF,* asparagine synthase. DHAP dihydroxyacetone phosphate; Gly3P Glycerol 3-phosphate; G6P glucose 6-phosphate; F16BP fructose 1,6-bisphosphate; GAP glyceraldehyde 3-phosphate; F6P fructose 6-phosphate; 6PGNL 6-phosphogluconolactone; R5P ribose 5-phosphate; Ru5P ribulose 5-phosphate; S7P sedoheptulose 7-phosphate; Xu5P xylulose 5-phosphate; PEP phosphoenolpyruvate; E4P erythrose 4-phosphate; ACoA acetyl-coenzyme A; PYR pyruvate; OAA oxaloacetate; CIT citrate; DHQ 3-dehydroquinic acid; DAHP 3-deoxy-Darabinoheptulosonate7-phosphate; QA quinic acid; DHS 3-dehydroshikimic acid; SA shikimic acid; GA gallic acid; CHO chorismate.

In recent years, the use of renewable resources to produce chemicals and fuels has attracted the attention of researchers^9–11^. Different than other aromatic compounds, DHHA can be synthesized in microorganisms. McCormick et al. first isolated DHHA from the fermentation broth of *Streptomyces aureofaciens* S-652 and Meade et al. obtained an *S. aureofaciens* strain which had a production of 8 g/L of DHHA after 120 h of fermentation^3,12^. According to previous research by Mavrodi et al., benzoate was converted to 2-amino-4-deoxycholic acid (ADIC) by PhzE, and then converted by PhzD to DHHA, in certain *Pseudomonas* strains*.* DHHA is an important intermediate of the phenazine biosynthesis of *Pseudomonas spp* (Fig. 1)^13,14^.

Lzh-T5 is a *P. chlororaphis* strain isolated from the tomato rhizosphere located in China. It has a phenazine biosynthesis cluster *phzABCDEFG*, and could produce phenazine-1-carboxylic acid (PCA) and other phenazine derivatives^15^. In this study, *phzF* of *P. chlororaphis* Lzh-T5 was disrupted causing DHHA accumulation. Three negative regulatory genes (*pykF*, *psrA,* and *rpeA*) were stepwise disrupted in *P. chlororaphis* Lzh-T5. The yield of DHHA increased from 2.15g/L to 5.52 g/L. To improve DHHA production, key genes were overexpressed by BglBrick vectors from the shikimate, pentose phosphate, and gluconeogenesis pathways of Lzh-T5, obtaining 7.89 g/L DHHA. The effect of Fe^3+^ on DHHA production was investigated. A final DHHA yield of 10.45 g/L was obtained.

## Methods

### Microorganisms, growth conditions, and plasmids

All plasmids, primers, and microorganisms are listed in Table 1 and Supplementary Material 1: Table S1. All *Escherichia coli* strains were cultured in LB medium at 37 °C*,* and all *P. chlororaphis* strains were cultured in KB medium at 30 °C. Kanamycin and Ampicillin were used. All details are detailed in our previous research^16^.

**Table 1:** Strains and plasmids used in this study.

Strains and plasmids	Relevant gene type	Reference/source
**Strains**		
DH5α	*E. coli* F^−^Ф80*lacZ*ΔM15 Δ(*lacZYA*-*argF*) U169 *recA1 endA1 hsdR17* (r_k_^−^ m_k_^−^) *phoA supE44 thi*^−1^ *gyrA96* relA1	Lab stock
*E.coli* S17-1(λpir)	res^−^ pro mod^+^ integrated copy of RP4, mob^+^, used for incorporating constructs into *P. chlororaphis*	Lab stock
Lzh-T5	wild-type strain of *P. chlororaphis* Lzh-T5	Lab stock
LDA-1	*phzF* gene deletion of *P. chlororaphis* Lzh-T5	This work
LDA-2	*pykF* gene deletion of *P. chlororaphis* LDA-1	This work
LDA-3	*psrA* gene deletion of P. chlororaphis LDA-2	This work
LDA-4	*rpeA* gene deletion of *P. chlororaphis* LDA-3	This work
LDA-5	*aroE, aroD, aroB, phzC, tktA and ppsA overexpression in* LDA-4	This work
**Plasmids**		
pEASY-Blunt	Blunt vector of gene coloning , Ap^r^, Kan^r^	Lab stock
pEASY-Blunt-aroD	Site mutant vector of *aroD*	This work
pEASY-Blunt-tktA	Site mutant vector of *tktA*	This work
pEASY-Blunt-ppsA	Site mutant vector of *ppsA*	This work
pK18mobsacB	Broad-host-range gene replacement vector, *sacB*, Kan^r^	Lab stock
pK18-phzF	vector for *phzF* deletion	This work
pK18-pykF	vector for *pykF* deletion	This work
pK18-rpeA	Vector for *rpeA* deletion	This work
pK18-psrA	Vector for *psrA* deletion	This work
pBbB5K-GFP	pBBR1; Kn^r^ lacI P_lac-UV5_	Lab stock
pBbB5K-aroE-aroD-aroB-phzC-tktA-ppsA	plasmid for *aroE, aroD , aroB ,phzC, tktA* and *ppsA ,* cooverexpression	This work

### DNA manipulation

A no-scar deletion method was used in the genome of *P. chlororaphis* Lzh-T5 and its derivative strain. In order to create LDA-1 by the interruption of *phzF* in Lzh-T5, two pairs of primers (phzF-A (EcoRI)–phzF-B and phzF-C–phzF-D (XbaI)) were designed. Upstream (740 bp) and downstream (793 bp) of *phzF* were first amplified by PCR. A 1515 bp fragment fusion was amplified by overlap PCR. The fusion fragment was constructed into pK18mobsacB, creating the recombinant plasmid pK18-phzF.

The pK18-phzF plasmid was transferred to *E. coli* S17-1 (λpir) by heat shock transformation. Then biparental mating between *E. coli* S17-1 and *P. chlororaphis* Lzh-T5 generated the mutant LDA-1 strain. Single-crossover and double-crossover clones were selected stepwise. To ensure accuracy, PCR analysis and sequencing were used to confirm the deletion. Detailed steps are listed in our previous research^16^. Similarly, *rpeA*, *pykF*, and *psrA* were disrupted in their corresponding strains.

BglBrick plasmids are a kind of widely used Brick plasmids^17^. The plasmid pBbB5K-GFP was used as the backbone to overexpress six key genes. Recombinant plasmids used in this study were constructed following the methods described in our previous research^16^. In brief, six Brick plasmids containing *aroB*, *aroD*, *aroE*, *phzC*, *tktA,* and *ppsA,* respectively, were first constructed. Point mutations were made in order to remove restriction sites (EcoRI, HindIII, BamHI, and BglII) in *aroD*, *tktA,* and *ppsA*. Then, a complicated overexpression plasmid pBbB5K-aroE-aroD-aroB-phzC-tktA-ppsA was constructed following the BglBrick standard.

### Quantitative RT-PCR

Quantitative RT-PCR was used to detect the transcriptional changes of related genes of different strains. And the *rpoD* which is one of housekeeping gene in *P. chlororaphis* was selected as the internal reference gene. The measurement methods of Quantitative RT-PCR are following our previous work^18^. And the fold change for mRNAs was calculated by the 2^−ΔΔCt^ method^19^.

### Fermentation processing

All *P. chlororaphis* strains were stored in an ultra-low temperature refrigerator. Strains were activated in KB petriplate with Ampicillin at 30 °C for 12–24 h before fermentation. Single colonies were inoculated in a 50 mL flask which contains 5 mL of KB medium and incubated overnight. Portions of seed bacteria were then inoculated in 50 mL KB medium in a 250 mL baffled flask and inoculation initial OD600 was 0.03. The gene expression of BglBrick plasmids was induced by isopropyl-β-d-thiogalactopyranoside (IPTG). 50 μM IPTG was used during an incubation of 12 h. After growing at 30 °C and centrifuged at 200 rpm for 24–72 h, the fermentation broth was collected to measure phenazine compounds and OD600. All experiments were performed in triplicate, and data were averaged and reported as mean ± standard deviation. After growing at 30 °C and 200 centrifuged at rpm for 24–72 h, the culture was collected for measurement of phenazine compounds and OD600. All fermentation experiments were performed in triplicate, and the data were averaged and reported as mean ± standard deviation.

### Measurement, purification, and quantification of DHHA from fermentation broth

Determination and purification of DHHA from fermentation broth followed the methods described in previous research^20^. Briefly, the supernatant of the fermentation broth was collected by centrifuging at 12,000*g* for 15 min. Then, it was analyzed by liquid chromatography–mass spectrometry (LC–MS) after processing through a 0.22 μm polyvinylidene difluoride syringe filter. LC–MS was performed on the Agilent HPLC1290-MS6230 system (Agilent Technologies, Santa Clara, CA) by an Agilent Extend C18 column (50 mm × 2.1 mm, 1.8 μm). It was eluted with methanol/0.1% formic acid (50:50, v/v) at a 0.15 mL/min flow rate. The DHHA sample was analyzed by mass spectrometry in the positive ion detection mode.

In order to purify DHHA, Shimadzu Inert Sustain phenyl column (20 × 250 mm, 15 μm) was used in HPLC (Shimadzu LC8A, Shimadzu, Kyoto, Japan). It was eluted with water/methanol (90:10, v/v) with a flow rate of 2 mL/min at a scanning wavelength of 278 nm. We collected the peak containing DHHA and dried it by vacuum freezing. The crystals of DHHA were obtained at room temperature after dissolving them in hot ethanol (65 °C).

Quantification of DHHA was described in our previous work ^18^. Briefly, the fermentation broth was centrifuged at 11,000 rpm for 3 min and supernatant collected. The samples were analysis by HPLC (Agilent 1260, USA) with a Shimadzu Inert Sustain phenyl column (4.6 × 250 mm, 5 μm) to determine the amount of DHHA. It was eluted with 0.1% formic acid/methanol (85:15, v/v) with a flow rate of 1 mL/min at a scanning wavelength of 278 nm. A DHHA standard curve was used to infer the DHHA content by plotting the concentration of the DHHA standard solution on the abscissa and the corresponding absorbance (peak area) on the vertical.

### Superoxide dismutase activity measurement

In the Fe^3+^ addition experiments, superoxide dismutase (SOD) activities of different strains were measured. The methods of measure are following the previous literature^21,22^.

## Results

### Disruption of phenazine synthesis in Lzh-T5

**Figure 2:**
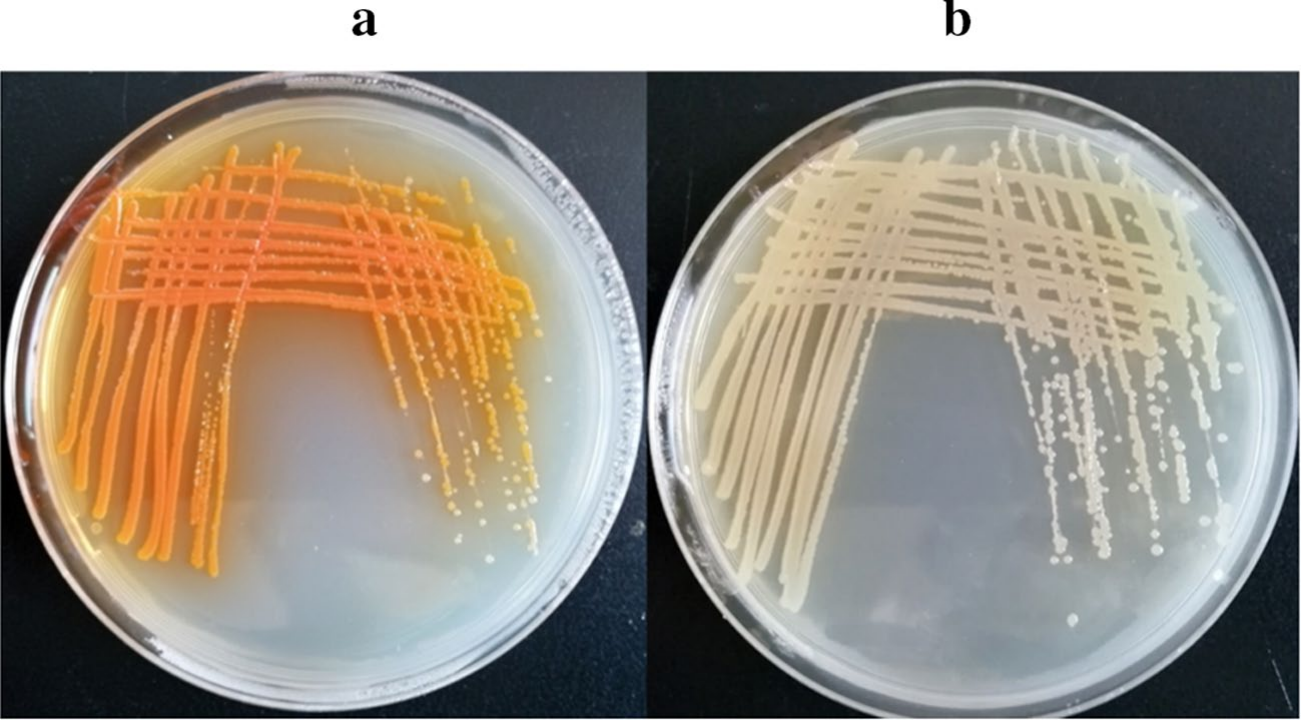
Colony morphology of *P. chlororaphis* Lzh-T5 and LDA-1. (**a**) Colony morphology of *P. chlororaphis* Lzh-T5. (**b**) Colony morphology of *P. chlororaphis* LDA-1.

**Figure 3:**
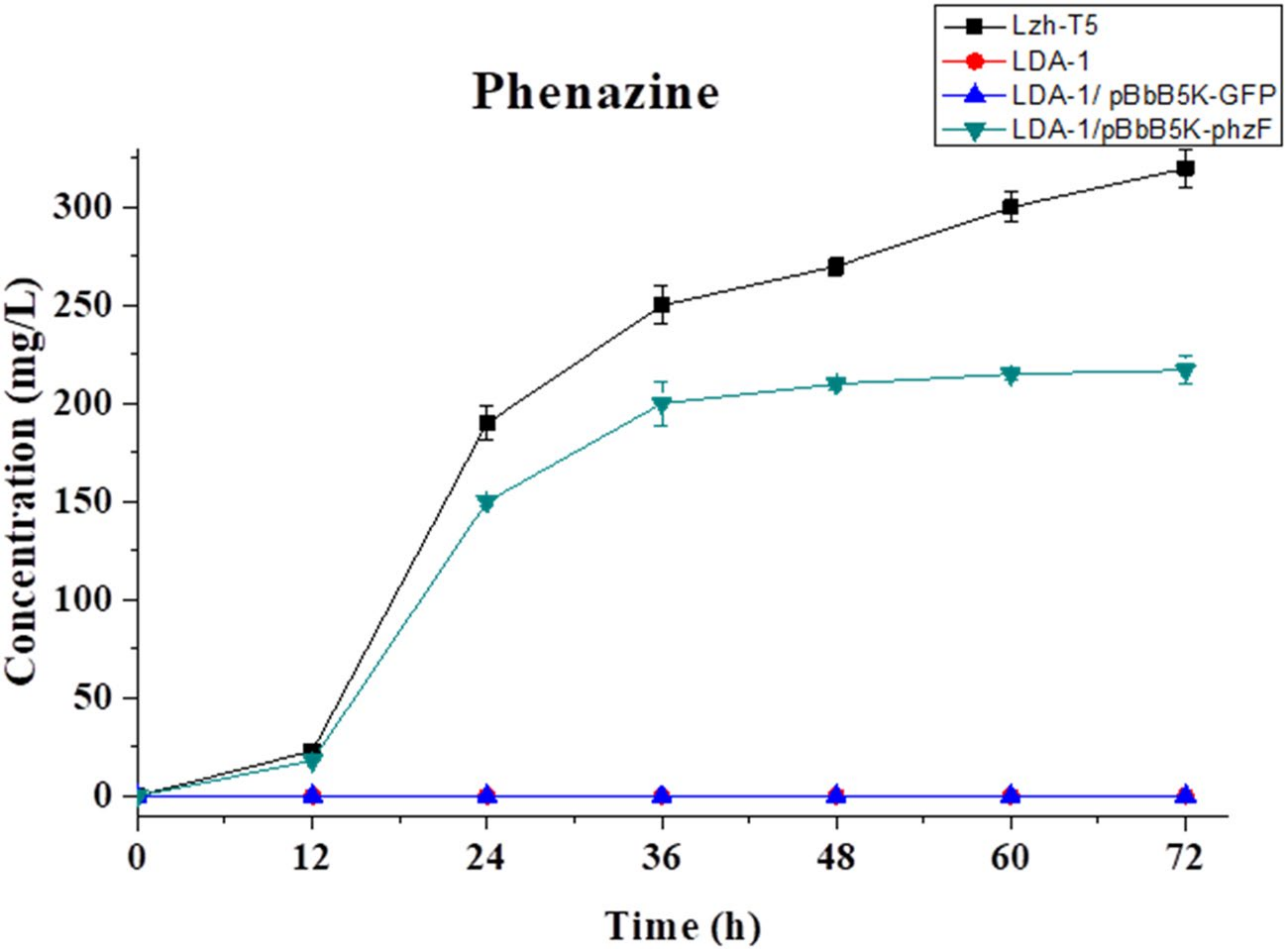
Phenazine production of *P. chlororaphis* Lzh-T5, LDA-1, LDA-1/pBbB5K-GFP, and LDA-1/pBbB5K-phzF.

According to the research from Blankenfeldt, DHHA undergoes an isomerization reaction facilitated by PhzF and converts to 6-amino-5-oxocyclohex-2-ene-1-carboxylic acid in *Pseudomonas* spp^23^. In order to accumulate DHHA, *phzF* was disrupted in *P. chlororaphi*s Lzh-T5, and the strain *P. chlororaphis* LDA-1 was got. As shown in Fig. 2, after 48 h of culture, the *P. chlororaphis* LDA-1 strain on an agar plate turns milky; however, the colony color of Lzh-T5 remains orange. This suggests that the phenazine derivatives could not be synthesized in LDA-1. After fermentation and analysis by HPLC–UV, phenazines (including 2-hydroxyphenazine, PCA, and 2-OH-PCA) disappeared in the broth of *P. chlororaphis* LDA-1 (Fig. 3).

**Figure 4:**
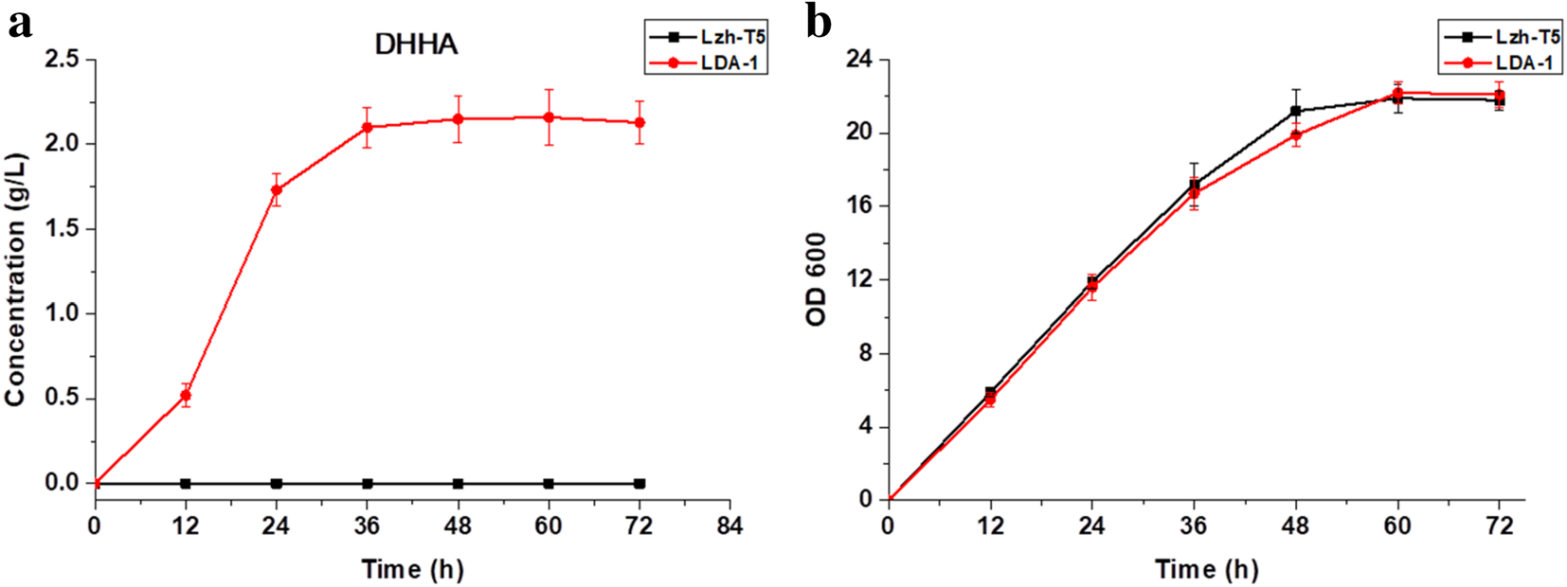
The DHHA production, growth curves of *P. chlororaphis* Lzh-T5 and LDA-1. (**a**) The DHHA production. (**b**) Growth curves.

Similar to previous research, a new absorption peak appears in the HPLC–UV at a wavelength of 278 nm, which is the maximum absorption wavelength of DHHA (Fig. S1)^20^. According to the analysis of LC–MS, the mass-to-charge ratio (m/z) of the compound was 156.0658 for [C^7^H_9_NO_3_^+^ H]^+^ (the mass of DHHA is 155.0655; Fig. S1). When *phzF* is overexpressed in the LDA-1 strain, production of phenazine was recovered (Fig. 3). According to these results, it suggests that disruption of *phzF* in Lzh-T5 causes accumulation of DHHA. Using HPLC–UV analysis, the DHHA yield of LDA-1 reached 2.15 g/L in 48 h (Fig. 4a). Our results suggest that disruption of *phzF* had little effect on Lzh-T5 cell growth (Fig. 4b).

### Knockout of negative regulatory genes to boost DHHA production

**Figure 5:**
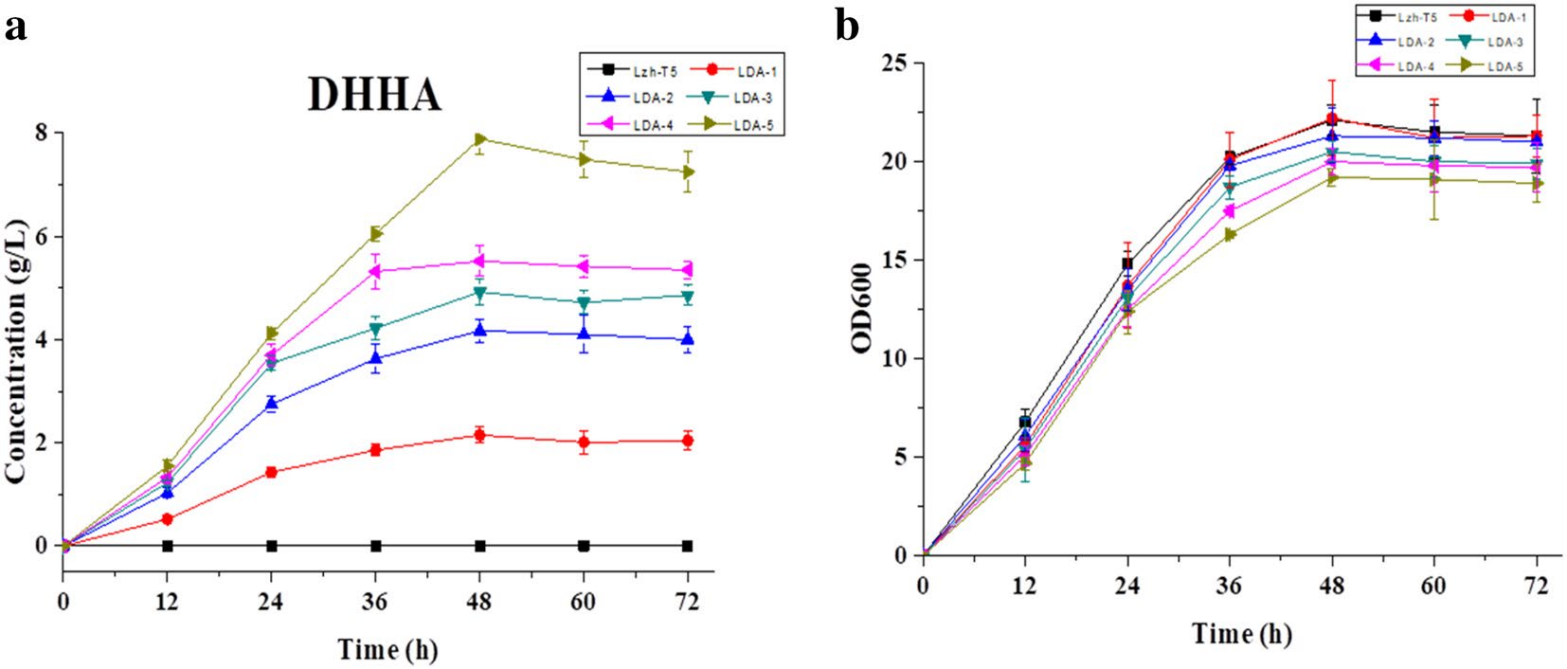
The DHHA production, growth curves of *P. chlororaphis* Lzh-T5, LDA-1, LDA-2, LDA-3, LDA-4, and LDA-5. (**a**) The DHHA production. (**b**) Growth curves.

According to previous research, interruption the *pykF* (which encode pyruvate kinase) enhances the production of 2-hydroxyphenazine^16^. In this study, *pykF* from LDA-1 was initially chosen to inactivate, so we obtained a mutant strain LDA-2 (Fig. 5). After analysis of the fermentation broth by HPLC–UV, the DHHA yield of strain LDA-2 increased from 2.15 g/L to 4.17 g/L (Fig. 5a**).** The growth condition of the strain was detected to have little effect after deletion of *pykF* (Fig. 5b**).**

PsrA, a sigma regulator, was first reported in *P. chlororaphis* PCL1391^24^. According to Chin-A-Woeng et al., PsrA played a negative regulatory role in the production of the antifungal metabolite PCA in *P. chlororaphis* PCL1391^24^. Similar results were obtained from *psrA* disruption in *P. chlororaphis* HT66 (Peng et al. 2018)^25^. According to our research, the sigma regulator *psrA* also exists in *P. chlororaphis* Lzh-T5. The strain LDA-3 was obtained after the gene *psrA* was disrupted in LDA-2, and the production of DHHA increased from 4.17 g/L to 4.92 g/L (Fig. 5a).

RpeA, a negative regulator of Phenazine, was mutated by insertion in *P. chlororaphis* GP72 and results in a production increase of 2-hydroxyphenazine^16,26^. RpeA is part of the two-component signal transduction system (TCST) RpeA/RpeB, and is present in other *Pseudomonas* strains. For example, in *P. chlororaphis* 30–84, an RpeA homologue, negatively regulates the yield of PCA, indicating a conserved mechanism of *Pseudomonas spp* in phenazine synthesis regulation^27,28^. In this study, *rpeA* was disrupted in the LDA-3 genome to construct LDA-4. Similar to the insertional mutagenesis of *P. chlororaphis* GP72, DHHA yield of LDA-4 increased from 4.92 g/L to 5.52 g/L (Fig. 5a). After knocking out the negative regulatory genes, quantitative RT-PCR results showed that the transcript level of genes *phzD* and *phzE* in the derivative strains, which is key gene for DHHA synthesis, has increased significantly (Fig. S2).

### Enhanced DHHA production by key gene overexpression

We disrupted *pykF* to improve the yield of DHHA from 2.15 g/L to 4.17 g/L by diverting more metabolic flux into the shikimate pathway from other pathways (Fig. 5a). This indicates that enhancing the lead synthesis pathway, we could improve the yield of DHHA in *P. chlororaphis* Lzh-T5. Compared with knocking out negative regulatory genes, gene overexpression is another effective strategy often used to increase the yield of biologic products in microorganisms. According to previous research, overexpression of key genes in the shikimate pathway enhanced the yield of 2-OH-PHZ^16^. In order to increase the production of DHHA, *aroB*, *aroD*, *aroE*, *phzC*, *tktA*, and *ppsA* from Lzh-T5 were overexpressed from the shikimate, pentose phosphate, and gluconeogenesis pathways. We used a previously employed kind of modular vector, the BglBrick plasmid^29^. A recombinant plasmid containing six genes, pBbB5K-aroE-aroD-aroB-phzC-tktA-ppsA, was constructed. Strain LDA-5 was created after transformation into the strain LDA-4 by electrotransformation. After fermentation, the DHHA production of LDA-5 increased to 7.89 g/L after 48 h (Fig. 5a). This indicates that overexpression of key genes is an effective strategy to enhance the production of DHHA. Quantitative RT-PCR results showed that the transcript level of six genes overexpressed in the derivative strain LDA-5 has increased (Fig. S3).

### Enhanced DHHA production with Fe^3+^

**Figure 6:**
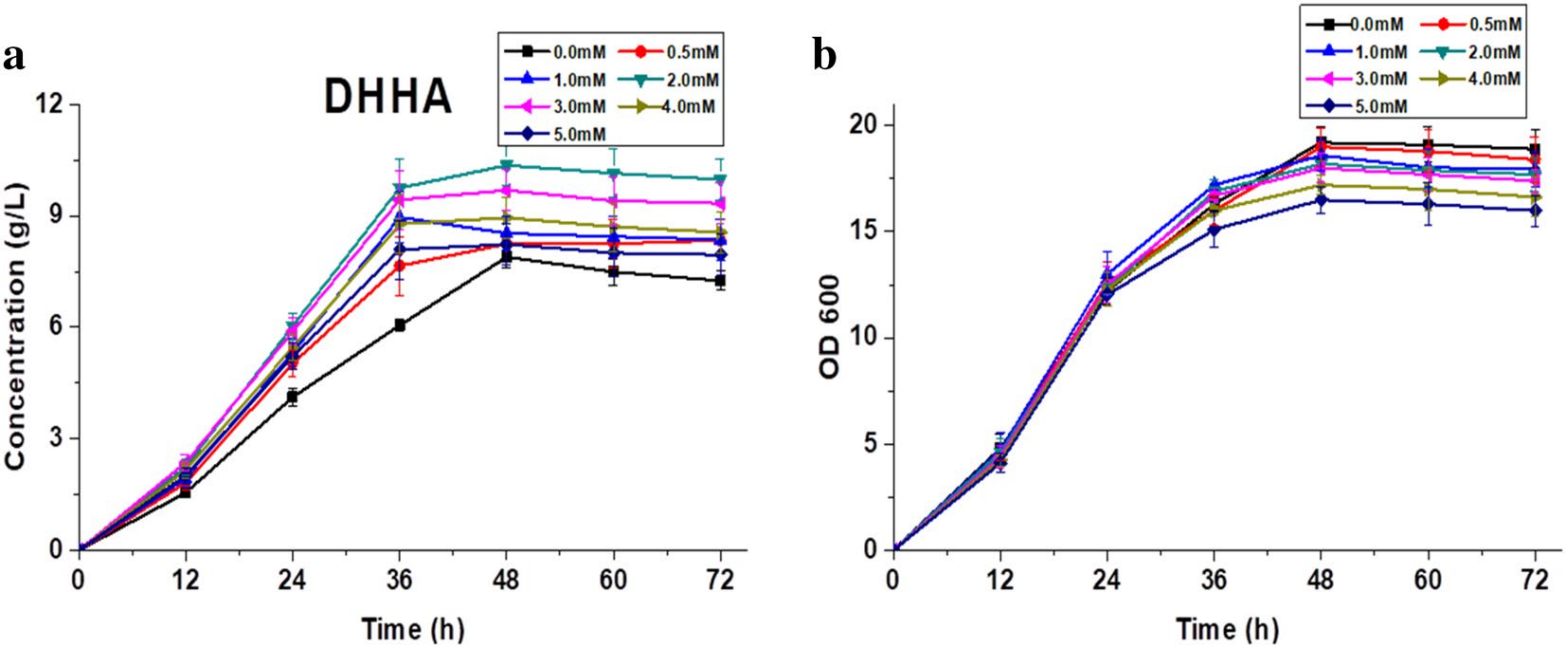
The DHHA production, growth curves of *P. chlororaphis* LDA-5 after different concentrations of Fe^3+^ adding. (**a**) The DHHA production and (b) growth curves.

Environmental factors have important effects on secondary metabolite production in *Pseudomonas* strains, especial ion concentration in the medium^30,31^. There is no universal medium suitable for *Pseudomonas* strains which can produce phenazines due to different nutritional requirements^30^. According to previous research, the DHHA production has a 30% increase after adding 3 mM of Fe^3+^^20^. To improve the production of DHHA, the effect of different concentrations of Fe^3+^ in the medium was investigated. After fermentation, DHHA production was detected by HPLC–UV. Low concentration of iron ions promoted DHHA production. High concentrations of iron ions inhibited DHHA production (Fig. 6). Different from our previous research, in the fermentation of LDA-5, the optimum concentration which enhanced the production of DHHA was 2 mM. We obtained a maximum DHHA yield of 10.45 g/L with 2 mM Fe^3+^ (Fig. 6a).

## Discussion

*Pseudomonas spp* is a class of microorganism that exists widely in the environment^32^. It has strong adaptability and often produces resistant substances. Among these substances, phenazine derivatives are typical secondary metabolites, such as PCA, Pyocyanin (PYO), 2-Hydroxyphenazine and phenazine-1-carboxamide (PCN)^25,33^. Phenazine derivatives are produced by the phz gene clusters (*phzABCDEFG*) found in several *Pseudomonas spp* (including *P. chlororaphis, P. fluorescence,* and *P. aeruginosa*)^34–36^. In *P. chlororaphis*, phzC catalyzes the first reaction of the shikimic acid pathway, by catalysis of E4P and PEP to synthesize DAHP^13^. PhzE, the first key enzyme in the phenazine synthesis pathway which catalyzes the last product of the shikimate pathway into 2-amino-4-deoxy branched acid^37^. ADIC converts to DHHA and pyruvate by the isobranse enzyme PhzD^23^. It is then epimerized with diaminopimelate enzyme (diaminopimelate epimerase (DapF)) PhzF to 6-amino-5-oxocyclohex-2-ene-1-carboxylic acid (AOCHC)^38^. Two molecules of AOCHC are converted to hexahydrophenazine-1,6-dicarboxylic acid (HHPDC) catalyzed by the dimer PhzAB^23^. HHPDC spontaneously undergoes oxidative decarboxylation to form tetrahydrophenazine-1-carboxylic acid (THPCA). THPCA is catalyzed by PhzG to form 5,10-dihydro-phenazine-1-carboxylic acid (DHCCA)^39^, DHCCA eventually undergoes self-oxidation in the air to form PCA. While DHHA is an important intermediate product of phenazines, the production of phenazines is quite low. For example, in wild type *P. chlororaphis* GP72, the yield of 2-OH-PHZ is 4.5 mg/L, and the higher phenazine (PCN) production in wild type is about 400 mg/L in *P. chlororaphis* HT66^25,26^. The phenazine production strains are also the potential DHHA producers. *P. aeruginosa* is an opportunistic pathogenic bacterium, while *P. chlororaphis* is not. *P. chlororaphis* was selected as a candidate for DHHA production^16^. According to previous research, *P. chlororaphis* GP72 could accumulate 1.92 g/L DHHA with *phzF* disruption^20^. Lzh-T5 is a *P. chlororaphis* strain isolated from the tomato rhizosphere found in China. It has the phenazine biosynthesis cluster *phzABCDEFG*, and can produce phenazine derivatives^15^. In this study, *phzF* was disrupted from the genome of *P. chlororaphis* Lzh-T5 making the strain *P. chlororaphis* LDA-1 which has 2.15 g/L DHHA accumulation.

Enhancing the shikimate pathway is an effective strategy for aromatic compound production in microorganisms^20^. The direct precursors of the shikimate pathway are PEP and E4P. The PEP and E4P supply could be enhanced by an overexpression of PEP synthase encoded by *ppsA* and transketolase encoded by *tktA*^1,40^. In addition, inactive *pykF,* which encodes pyruvate kinase, can increase PEP^41,42^. Further carbon flux of the shikimate pathway impedes enzymatic reactions and removes the allosteric and transcriptional regions^20,43^. Quinate/shikimate dehydrogenase, dehydroquinic acid synthase, dehydroquinic acid dehydratase, and DHAP synthetase, encoded by *aroE*, *aroB*, *aroD,* and *phzC,* respectively, were reported as limiting steps in the shikimate pathway^44^. Our previous studies showed that disruption of pyruvate kinase (encoded by *pykF*) and coexpression of *ppsA*, *tktA*, *aroB*, *aroD*, *aroE*, and *phzC* in GP72 increased phenazine production^20^. In this study, the disruption of *pykF* and six gene *tktA*, *ppsA*, *aroB*, *aroD*, *aroE*, and *phzC* coexpression in LDA-1 increased the DHHA production to 7.89 g/L.

TCST systems exist in *Pseudomonas spp* that help them adapt to the environment by coordinating cellular pathways to interact with the environment^28^. Different TCST systems were found in *P. chlororaphis* Lzh-T5. Among these TCST systems, GacS/GacA is one of the most researched. GacS/GacA was the earliest TCST system which could enhance bioactive secondary metabolite production in *Pseudomonas spp*^45,46^. In *P. chlororaphis* HT66, GacA positively regulates the expression of *psrA*. *psrA* negatively controls the expression of *rpoS* and the expression of phenazine^25^. In this study, *psrA* was found in *P. chlororaphis* Lzh-T5. Interruption of *psrA* increased the production of DHHA from 4.17 g/L to 4.92 g/L, this suggests that *psrA* negatively controls the production of DHHA. RpeA/RpeB is a TCST system found widely in *Pseudomonas spp*^28^. According to the research of Whistler et al., Pip improved the production of PCA by enhancing the expression of *phzR* and *phzI*. Sigma factor *rpoS* regulated the expression of *pip*, which itself is regulated by the RpeA/RpeB TCST system. RpeB was inhibited by RpeA, so an *rpeA* mutant strain enhanced the production of phenazine^28^. Similar results were observed from *P. chlororaphis* GP72 and *P. chlororaphis* HT66^16,25^. The rpeA/rpeB TCST system was found in *P. chlororaphis* Lzh-T5, interruption of *rpeA* results in the increase in DHHA from 4.92 g/L to 5.52 g/L (Fig. 5a). This result suggests that, similar with *P. chlororaphis* 30–84, *rpeA* negatively impacts the production of DHHA.

Metal ions are an important factor which could affect the production of secondary metabolites of *Pseudomonas spp*^30^. Fe^3 +^ is one of the important ions, and according to the research of Shtark et al., it plays a positive role in the production of phenazines in *P. chlororaphis* SPB1217. They hypothesize that Fe^3+^ activates some dependent superoxide dismutases and those superoxide dismutases promotes phenazine production by removing reactive oxygen species or bacterial metabolites that suppress enzymes involved in the synthesis pathway of phenazines^30^. Similar results were reported in *P. aeruginosa* PCL1391 and *P. fluorescens* 2–79^47,48^. Fe^3+^ also had a positive effect on DHHA production^20^. And our results shown that the SOD activity of the strain has increased after Fe^3+^ adding to the medium (Fig. S4). In this study, adding different concentration of Fe^3+^ had different effects on DHHA production in *P. chlororaphis* LDA-5. Low concentrations of Fe^3+^ promoted DHHA production, while high concentrations of Fe^3+^ inhibited DHHA production (Fig. 6). Adding 2 mM of Fe^3+^ had a positive effect on DHHA production, and our maximum DHHA yield was 10.45 g/L in this study.

In conclusion, *phzF* of *P. chlororaphis* Lzh-T5 was disrupted to construct the strain *P. chlororaphis* LDA-1 which had DHHA accumulation. Then, three negative regulatory genes (*pykF*, *psrA* and *rpeA*) were disrupted stepwise in *P. chlororaphis* LDA-1. The production of DHHA increased from 2.15 g/L to 5.52 g/L. Next, different key genes selected from the shikimate, pentose phosphate, and gluconeogenesis pathways of Lzh-T5, were overexpressed by BglBrick vectors. The production of DHHA increased from 5.52 g/L to 7.89 g/L. The effect of adding Fe^3+^ on DHHA production was investigated in a strain (LDA-5) with a resulting DHHA yield of 10.45 g/L.

## Supplementary Information

Supplementary Information.

## Data Availability

All data generated or analyzed during this study are included in this published article (and its Additional file).
