# Supplementary material for: Genetic engineering of Pseudomonas chlororaphis Lzh-T5 to enhance production of trans-2,3-dihydro-3-hydroxyanthranilic acid
Source: Sci Rep. 2021 Aug 12;11:16451. doi: 10.1038/s41598-021-94674-8 (PMC8361184; doi:10.1038/s41598-021-94674-8)
Supplement: Supplementary file 1 — Supplementary Information. [file 41598_2021_94674_MOESM1_ESM.pdf]

Scientific Reports

Genetic engineering of *Pseudomonas chlororaphis* Lzh-T5 to enhance production of trans-2,3-dihydro-3-hydroxyanthranilic acid

Kaiquan Liu[1#], Ling Li[1,2#]*, Wentao Yao[1], Wei Wang[3]*, Yujie Huang[2], Ruiming Wang[1],Piwu Li[1]

1State Key Laboratory of Biobased Material and Green Papermaking (LBMP), School of Bioengineering, Qilu University of Technology(Shandong Academy of Sciences), Jinan, Shandong, 250353, People's Republic of China.

2Shandong Provincial Key Laboratory of Applied Microbiology, Ecology Institute, Qilu University of Technology (Shandong Academy of Sciences), Ji'nan, 250103, People's Republic of China.

3State Key Laboratory of Microbial Metabolism, School of Life Sciences and Biotechnology, Shanghai Jiao Tong University, Shanghai, 200240, People's Republic of China.

# Co-first authors have contributed equally to this work

* Correspondence author:

The First corresponding author: Ling Li, E-mail: liling33802400@qlu.edu.cn

The Second corresponding author: Wei Wang, E-mail: weiwang100@sjtu.edu.cn

Figure S1. Identification of DHHA by LC-MS

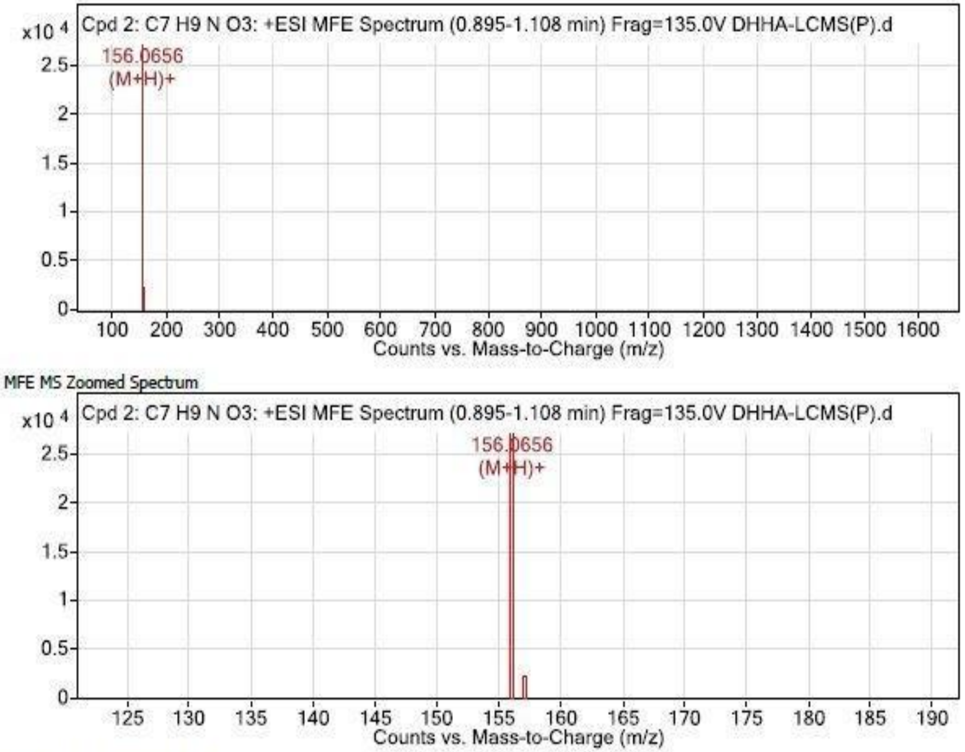

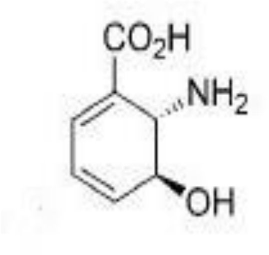

DHHA Structure

MS Spectrum Peak List

m/z	z	Abund	Formula	Ion
156.0656	1	27130.4	C7 H10 N O3	(M+H)+
157.0693	1	2412.9	C7 H10 N O3	(M+H)+

Predicted Isotope Match Table

Isotope	*m/z*	Calc m/z	Diff (ppm)	Abund %	Calc Abund %	Abund Sum %	Calc Abund Sum %
1	156.0656	156.0655	-0.29	100	100	91.83	92.45
2	157.0693	157.0686	-4.21	8.89	8.17	8.17	7.55

Table S1. Primers used in this work.

Primers	Sequence 5' → 3'	Application
phzF-A	CCGAATTCAAAGCCGGGCCAAGGCCACTGGCC	*phzF*gene deletion
phzF-B	CGTAGTTGTGCATGGTTATCTCCCGG	
phzF-C	ACCATGCACAACTACGATCGTTCTATGAACA	
phzF-D	ATAAGCTTAGCAGCCAGGCACTTCAAAGCATGT	
pykF-A	CACGAATTCCGCCGGTCCGTGAACATGTCAT	*pykA* gene deletion
pykF-B	GCAAAGACTCCTGAGTTCAAGCGCAACGAGAGG	
pykF-C	TCAGGAGTCTTTGCGTCCCCCTGATGCAAC	
pykF-D	AAGTCTAGACGAAGGACTGCGACAAGCCGACG	
psrA -A	GCGGGATCCGGTCCAAACCGACTGCCAACA	*psrA* gene deletion
psrA -B	CAGGTGCGTGAGCTGAGTGC	
psrA -C	ACTCAGCTCACGCACCTGATGGCTACTCCGCCTGACAAA	
psrA -D	CCCAAGCTTCGGATTGATGTTGCAGGACTCTTC	
rpeA-A	GCCAAGCTTCTCCAAGGAACGTTGTCGCTGTGTTT	*rpeA gene deletion*
rpeA-B	CGACTCAGCATTCCCATTCGGAACGGCTGAACA	
rpeA-C	AATGGGAATGCTGAGTCGTGTTCGAGCCTTTCTACCG	
rpeA-D	GCCTCTAGACTGGTCATCGACGTACAGAACGACTTC	
tktA-F	AAAATGCCAAGCCGTCGTGAGCGTG	*tktA* gene cloning
tktA-R	TTAGTCTTCCAGCAGCTCTTCAGCCTGA	
phzC-F	GGGGATGGAAGACTTACTGAAACGGG	*phzC* gene cloning
phzC-R	TCAAAAGGAGGCAAGGGTTGAGGTG	
aroB-F	ATGCAGACACTTAAGGTCGATCTAGGCGAG	*aroB* gene cloning
aroB-R	TTAACCTTTAAGCTGAGCCAGGGCGC	
aroD-F	ATGCGCCCTATCGTTCTGGTGCTC	*aroD* gene cloning
aroD-R	TCATGCCTGGGCTCCTTGAAAACG	
aroE-F	ATGGATCAGTACGTCGTTTTTGGTAACCCG	*aroE* gene cloning
aroE-R	ATATATCAGAGCCCCAGCTGGCGCC	
ppsA-F	CCCCTTGGTAGAGTACGTAGTTTCCCTC	*ppsA* gene cloning
ppsA-R	TTAGACCGCACCCTGCCCCTC	
phzC-F	GGGGATGGAAGACTTACTGAAACGGG	*phzC* gene cloning
phzC-R	TCAAAAGGAGGCAAGGGTTGAGGTG	
tktA-M1F	CGCCTACTCCGCCGA**G**TTCCCCGAGCT	First Site-directed mutagenesis for *tktA*
tktA-M1R	**C**TCGGCGGAGTAGGCGGAGAAGCGCTG	
tktA-M2F	CCCGCTGCTGCCGGA**G**TTCCTCGGC	Second Site-directed mutagenesis for *tktA*
tktA-M2R	**C**TCCGGCAGCAGCGGGCCGAACGC	
aroD-MF	GTGGCCGTTGCGCCGG**A**ATCCTGATCAACCCGG	Site-directed mutagenesis for *aroD*
aroD-MR	**T**CCGGCGCAACGGCCACGGGCGCC	

ppsA-M1F	GATCATGGAAGCCGA**G**TTCCCCGAGCAACTGAA	First Site-directed mutagenesis for *ppsA*
ppsA-M1R	**C**TCGGCTTCCATGATCCATTGACGGATCTGGGCG	
ppsA-M2F	TGACTGCTTCGAACT**T**GAGTGCCGCGCCCTC	Second Site-directed mutagenesis for *ppsA*
ppsA-M2R	CTCG**A**AGTTCGAAGCAGTCACGGAACGATTCGCTGATG	
ppsA-M3F	CCTGGCCGAAGAGTTCCT**T**GAGTTCTTCGACGG	Third Site-directed mutagenesis for *ppsA*
ppsA-M3R	**A**AGGAACTCTTCGGCCAGGATCGCGTTGGAAGG	
tktA--RB-F	AAGAATTCAAAAGATCTAAAAGGAGGCCATCCATGCCAAGCCGTCGT	*tktA* gene overexpression
tktA-RB-R	TACTCGAGTTTGGATCCTTAGTCTTCCAGCAGCTCTTCAG	
phzC-RB-F	AAGAATTCAAAAGATCTAAAAGGAGGCCATCCATGGAAGACTTACTGAAACG	*phzC* gene overexpression
phzC-RB-R	AACTCGAGTTTGGATCCTCAAAAGGAGGCAAGGGTT	
aroB-RB-F	AAGAATTCAAAAGATCTAAAAGGAGGCCATCCATGCAGACACTTAAG	*aroB* gene overexpression
aroB-RB-R	AACTCGAGTTTGGATCCTTAACCTTTAAGCTGAGCCAGG	
aroD-RB-F	AAGAATTCAAAAGATCTAAAAGGAGGCCATCCATGCGCCCTATCGTT	*aroD* gene overexpression
aroD-RB-R	AACTCGAGTTTGGATCCTCATGCCTGGGCTCCTT	
ppsA-RB-F	AAGAATTCAAAAGATCTAAAAGGAGGCCATCCTTGGTAGAGTACGTA	*ppsA* gene overexpression
ppsA-RB-R	AACTCGAGTTTGGATCCTTAGACCGCACCCTG	
aroE-RB-F	AAGAATTCAAAAGATCTAAAAGGAGGCCATCCATGGATCAGTACGTCGT	*aroE* gene overexpression
aroE-RB-R	AACTCGAGTTTGGATCCTCAGAGCCCCAGCTGGC	

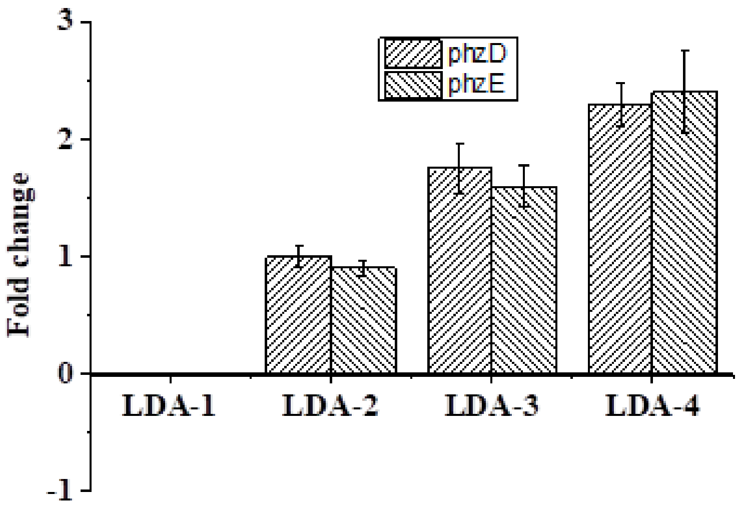

Figure S2 Transcriptional validation of key metabolic genes using Quantitative RT-PCR

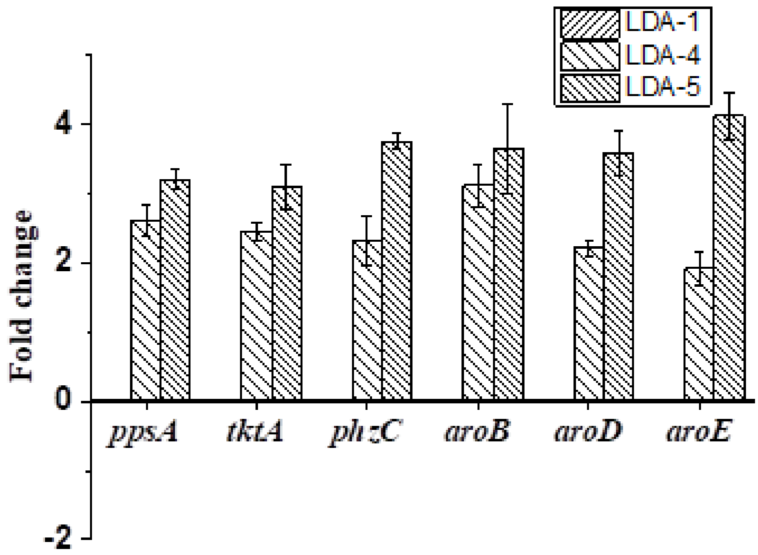

Figure S3. Transcriptional validation of six key gens of shikimic acid metabolism using Quantitative RT-PCR

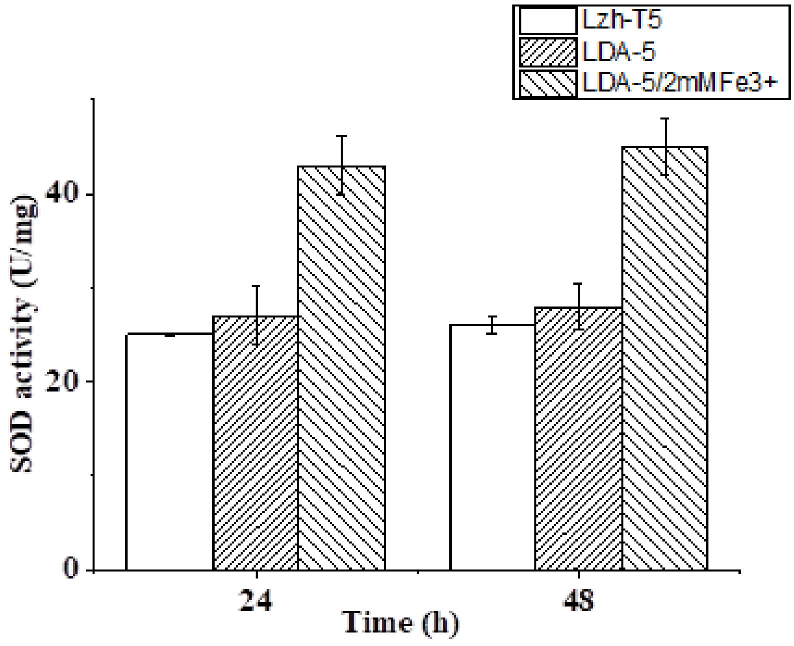

Figure S4. Superoxide dismutase (SOD) activity measurement of different strains
